# Epigenetic Determinants of Racial Disparity in Breast Cancer: Looking beyond Genetic Alterations

**DOI:** 10.3390/cancers14081903

**Published:** 2022-04-09

**Authors:** Shriya Joshi, Chakravarthy Garlapati, Ritu Aneja

**Affiliations:** 1 Department of Biology, Georgia State University, Atlanta, GA 30303, USA; sjoshi8@student.gsu.edu (S.J.); cgaralapati1@student.gsu.edu (C.G.); 2 Department of Clinical and Diagnostics Sciences, School of Health Professions, University of Alabama at Birmingham, Birmingham, AL 35294, USA

**Keywords:** breast cancer, racial disparity, epigenetic regulation

## Abstract

**Simple Summary:**

A substantial disparity in breast cancer incidence and mortality exists between African American (AA) and European American (EA) women. However, the basis for these disparities is poorly understood. In this article, we describe that gene–environment interactions mediated through epigenetic modifications may play a significant role in racial disparities in BC incidence and outcomes. Our in silico analyses and an in-depth literature survey suggest that there exists a significant difference in epigenetic patterns between AA and EA women with breast cancer. Herein, we describe the environmental factors that contribute to these epigenetic changes, which may underlie the disparate racial burden in patients with breast cancer. We suggest that AA women with higher basal epigenetic changes, may have higher pre-disposition to cancer onset, and an aggressive disease course. Pre-existing racial differences in epigenetic profiles of breast tissues raises the possibility of examining these profiles for early diagnosis.

**Abstract:**

Breast cancer (BC) is the most commonly diagnosed cancer in women. Despite advancements in BC screening, prevention, and treatment, BC incidence and mortality remain high among African American (AA) women. Compared with European American (EA) women, AA women tend to be diagnosed with more advanced and aggressive tumors and exhibit worse survival outcomes. Most studies investigating the determinants of racial disparities in BC have focused on genetic factors associated with African ancestry. However, various environmental and social stressors over an individual’s life course can also shape racial stratification in BC. These social and environmental exposures result in long-term changes in gene expression mediated by epigenetic mechanisms. Epigenetics is often portrayed as an intersection of socially patterned stress and genetic expression. The enduring nature of epigenetic changes makes them suitable for studying the effects of different environmental exposures over an individual’s life course on gene expression. The role of differential social and environmental exposures in racial disparities in BC suggests varied epigenetic profiles or signatures associated with specific BC subtypes in AA and EA women. These epigenetic profiles in EA and AA women could be used as biomarkers for early BC diagnosis and disease prognosis and may prove valuable for the development of targeted therapies for BC. This review article discusses the current state of knowledge regarding epigenetic differences between AA and EA women with BC. We also discuss the role of socio-environmental factors, including psychosocial stress, environmental toxicants, and dietary factors, in delineating the different epigenetic profiles in AA and EA patients with BC.

## 1. Introduction

Breast cancer (BC) is the second most common cause of cancer-related deaths among women in the United States [1,2,3,4]. Although a significant reduction in BC mortality has been achieved by increasing awareness and advancing BC diagnosis and treatment, a vast gap in BC mortality exists between African American (AA) and European American (EA) women. Studies have consistently shown higher BC mortality rates in AA women than in EA women. Although the overall BC incidence rate is slightly higher in EA women than in AA women, the BC incidence rate in women under the age of 45 is higher in AA women [5,6]. Furthermore, AA patients with BC exhibit more aggressive tumor phenotypes and worse overall survival than EA patients [1,2]. Racial and ethnic disparities in BC incidence and survival are significant public health concerns; however, the basis for these disparities is poorly understood.

**Figure 1:**
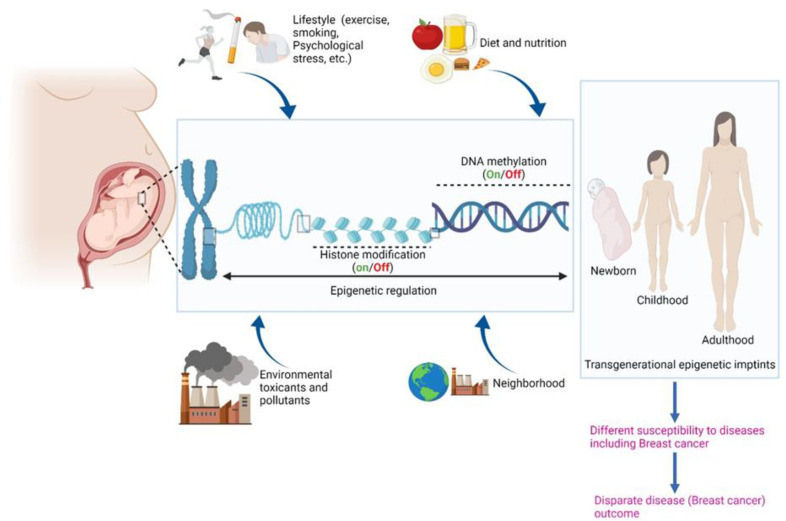
Schematic depicting role of epigenetics and various biological and non-biological factors in etiology of BC. Created with BioRender.com (accessed on 22 January 2022).

The reasons behind the racial disparities in BC incidence and outcomes are complex and multifactorial, involving both non-biological and biological factors. Gene–environment interactions play a substantial role in the development and progression of BC. Specifically, environmental factors, including pollutants, social stressors, and cultural and behavioral factors (e.g., dietary lifestyle and physical activity), play a pivotal role in the etiology of BC through modulation of gene expression. These factors also contribute to racial disparities in BC [7] (Figure 1).

Due to systemic inequalities, including structural racism, individuals in the US are exposed to different physical and social milieus through their life course according to their race or socioeconomic status [8]. These social determinants (i.e., structural inequities) play a significant role in disparities in health outcomes. A vast proportion of AAs is poorer than EAs, resulting in limited access to health care due to a lack of insurance or sufficient health coverage. Furthermore, low socioeconomic status is associated with behavioral risk factors, such as smoking, drinking, and physical inactivity, contributing to increased mortality [9]. AAs also face tremendous stress due to racial discrimination, which determines their access to housing and employment [10]. As a result of residential segregation along racial lines, AAs and minority communities tend to inhabit disadvantaged neighborhoods with high poverty, air pollution, and limited essential resources [11]. Factors associated with race and low socioeconomic status, including racial discrimination [12], neighborhood disadvantage, health behaviors, and exposure to pollutants [13], are associated with an increased risk of BC. Therefore, racial differences in social and environmental exposures over an individual’s lifetime can contribute to racial disparities in BC by influencing gene expression patterns. Structurally rooted biopsychosocial processes that the social patterning of stressors may activate in a historically unequal society could also influence cellular physiology and gene expression patterns in BC. In other words, gene–environment interactions mediated through epigenetic modifications may play a significant role in racial disparities in BC incidence and outcomes.

Epigenetic modifications (e.g., DNA methylation, histone modifications, and non-coding RNAs) can modulate gene expression without altering the DNA sequence (i.e., the inherited genome) [14]. Even though epigenetic modifications induced by environmental changes can be reversed, histone modifications and DNA methylation tend to be durable or transgenerational. As such, DNA methylation and histone modifications can serve as reliable molecular biomarkers to evaluate the cumulative effects of exposure to various physical and social environments over an individual’s life course. Lifetime exposure to specific environments associated with socioeconomic position and race may differentially imprint the epigenome, producing exposure-specific epigenetic signatures [15]. Consistent with this, specific DNA methylation patterns have been associated with low socioeconomic status, neighborhood disadvantage, and exposure to stressors [16]. In this review, we discuss the current state of knowledge about the differences in epigenetic patterns between AA and EA patients with BC. We also describe the environmental factors that contribute to these epigenetic changes.

## 2. Epigenome: A Tutor of the Genome

**Figure 2:**
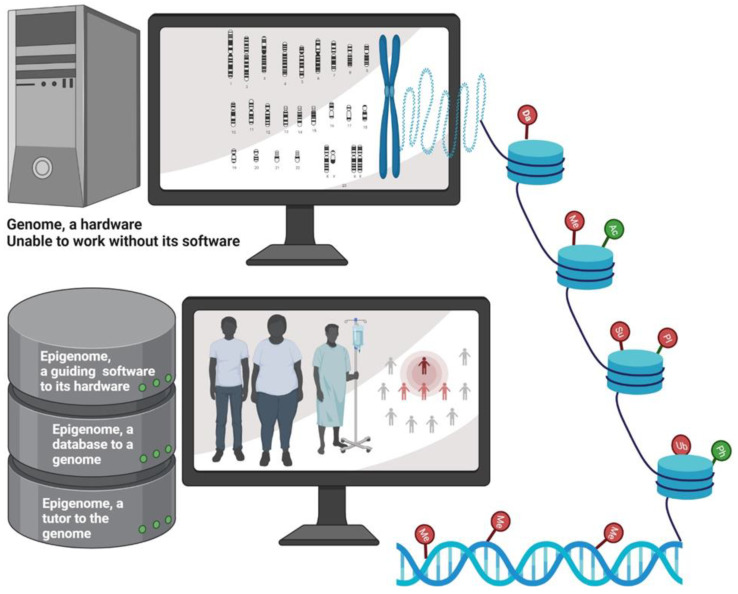
Schematic depicting epigenetics as a software which guides the genome. Epigenetic regulation determines the phenotype of the person. Epigenetic imprints are transgenerational and impacts a group of population. Da: deamination; Me: methylation; Ub: ubiquitination; Su: Sumoylation; PI: protein isomerization; Ph: phosphorylation; Ac: acetylation. Symbols represented in green indicate activation of target gene due to epigenetic modification, while those represented in red indicate repression of target gene upon epigenetic modification. Created with BioRender.com (accessed on 22 January 2022).

If we consider genes as the body’s hardware, then the epigenome is the software that instructs them how to work. Essentially, the epigenome is the instruction guide on top of the genome, and it serves as a dimmer switch that promotes a gene to turn on slightly, turn on fully, or turn off completely [17] (Figure 2). BC is a collusion of genetic and epigenetic alterations. Genetic and epigenetic changes intertwine and take advantage of each other during the BC initiation and progression, ultimately leading to aberrant gene expression. Changes in epigenetic regulators acquire genetic mutations, and genetic mutations in epigenetic regulators lead to an altered epigenome. It is believed that genetic changes acquired during BC progression are somewhat straightforward, but epigenetic pathway is much more intricate, which involves genome-wide loss of DNA methylation (hypomethylation), frequent hypermethylation in CpG islands of the promoter, changes in nucleosome occupancy, and modification profiles. Recent whole exome sequencing of various cancers including BC revealed that mutations in genes that regulate the epigenome are remarkably widespread [18]. Given that the epigenome works at the apex of the pyramid of gene control mechanisms, this suggests that the mutations in genes regulating the epigenome probably have impacts on multiple pathways of BC. Thus, in-depth research into how epigenetic and genetic modifiers communicate with each other to alter the expression is warranted.

Given the notion that genes are governed by biological memories of experiences acquired earlier in life or inherited by recent ancestors, studying epigenetic modifications serves as a potent alternative to the simple model of genetic determinism. Although genetic divergence between different ethnic groups could result in health disparities, evidence supports that BC inequalities are strongly influenced by various environmental stressors rather than being driven by genetic factors alone [19].

Studies have shown that social models of racial group identity might predict an individuals’ health status rather than genetic ancestry. Thus, comparing the experiences (e.g., social, physical, and nutritional experiences) of the present generation with those of their ancestors to identify epigenetic imprints may provide further insight into racial disparities in BC. Thus, a deep understanding of how genetic factors and the socially and economically structured environments that we inhabit interact to influence patterns in racially diverse BC patients is an unmet need.

## 3. Differences in DNA Methylation Patterns May Underpin Racial Disparity in Breast Cancer

Specific epigenetic changes involving DNA methylation and histone modifications have been associated with BC development and progression [20]. Various findings suggest that different epigenetic signatures may exist in AA and EA patients with BC. The presence of such epigenetic signatures is supported by recent studies demonstrating different methylation patterns in BC tissues from AA and EA patients [21]. Identifying epigenetic signatures and the corresponding environmental factors associated with these epigenetic patterns in AA and EA patients with BC will improve our understanding of BC etiology. Additionally, epigenetic patterns at specific sites may serve as diagnostic or prognostic biomarkers in AA and EA patients and guide the development of personalized therapies for different BC subtypes.

Due to its potentially enduring effects, DNA methylation is one of the most commonly studied epigenetic modifications in the context of cancer disparities [21]. In humans, DNA methylation typically occurs at cytosine nucleotides that are immediately followed by a guanine nucleotide (i.e., CpG dinucleotides). CpG dinucleotides are frequently found in the form of CpG repeats in the 5′ regulatory region of genes. These CpG repeats are known as CpG islands, and their methylation can modulate gene transcription. Although DNA methylation can either induce or suppress their expression, hypermethylation is often associated with gene silencing. Similarly, hypomethylation is associated with increased gene expression [22]. Aberrant methylation of CpG islands is a frequent and early event in BC [22]. Additionally, the methylation status of CpG islands is emerging as a diagnostic biomarker for BC [22].

Repression of tumor suppressor gene (TSG) expression has been observed in various cancers and is associated with poor prognosis [23]. Silencing of TSGs in cancer is often caused by hypermethylation of CpG islands in their promoter regions. Interestingly, differences in DNA methylation profiles of various TSGs have been observed between AA and EA patients with BC (Table 1) [21]. Mehrotra et al. investigated the methylation status of five TSGs (*SCGB3A1*, *TWIST1*, *CCND2*, *RARB*, and *RASSF1*) in AA and EA patients with BC [21]. *SCGB3A*, *TWIST1*, *CCND2*, and *RASSF1* were hypermethylated in AA but not in EA patients with BC. These racial differences in methylation patterns were more pronounced in patients under the age of 50 with ER−/PR− tumors than in younger patients with ER+/PR+ tumors [21]. TSG hypermethylation in AAs with ER−/PR− BC may contribute to the aggressive tumor phenotypes observed in AA women. Wang et al. showed that the TSG *CDH13* (encoding cadherin 13) was differentially methylated between AA and EA women with ER- BC [24]. Nevertheless, *CDH13* was not differentially methylated between AA and EA patients with ER+ BC [24].

Other cancer-related genes are also differentially methylated between AAs and EAs with BC. Conway et al. compared samples from AA and EA patients with BC and identified differential methylation patterns in several cancer-related genes, including *DSC2*, *KCNK4*, *GSTM1*, *AXL*, *DNAJC15*, *SNORD115*, *TUSC3*, and *TES* [27]. *TES* was hypermethylated in AA patients regardless of tumor type, whereas *TUSC3* was hypermethylated only in AA patients with hormone receptor-negative BC [27]. These findings indicate that the differential methylation of TSGs and other cancer-related genes between AA and EA patients with BC is strongly associated with BC subtype, receptor status, and patient’s age. Genes involved in transcriptional regulation, metabolism, and signal transduction have also been found to be differentially methylated between BC patients and healthy individuals (Table 2 and Table 3) [28]. Whether these genes also display racial discrepancies in their methylation status merits further investigation.

In a genome-wide DNA methylation profiling study, Ambrosone et al. identified 157 CpG sites that were differentially methylated in normal breast, ER− BC, and ER+ BC tissues from EA and AA women [29]. Almost twice as many CpG sites were differentially methylated (EA vs. AA) in ER- tumors, indicating that different etiological pathways may be involved in the development of ER− BC in AA and EA women. Racial differences in gene methylation patterns have also been observed in breast tissues from healthy women. In breast tissues from healthy AA and EA women, Song et al. identified 485 differentially methylated genes, including *AHRR*, *OPCML*, *PACS2*, HIPK2, and *TNK2* [30].

**Table 2:** Hypermethylated TSGs in patients with BC (reported data, race wise difference in methylation pattern not studied).

Tumor Suppressor Genes	Regular Function	Effect after Hypermethylation	Comments	References
***BRCA1***	▪ Tumor suppressor	▪ Increase in metastasis	▪ Hypermethylation in BC patients	[31]
***GSTP1***	▪ Increases chemosensitivity of drugs ▪ Detoxifying agent	▪ Increases BC risk ▪ Increases invasion and metastasis	▪ Hypermethylation in BC patients	[32]
***NKX-2*** ***NKX-5***	▪ Tumor suppressor	▪ Poor outcome in BC	▪ Hypermethylation in BC patients	[33]
***GARL2***	▪ Inhibits cell proliferation and induces apoptosis	▪ Poor prognosis in BC	▪ Hypermethylation in BC patients	[33]
***CDKN2A***	▪ Tumor suppressor	▪ Poor prognosis in BC	▪ Hypermethylation in BC patients	[34]
***RIL***	▪ Tumor suppressor	▪ Increase in tumor size, cell proliferation	▪ Hypermethylation in BC patients	[35]
***PTEN***	▪ Tumor suppressor	▪ Poor prognosis in BC	▪ Hypermethylation in BC patients	[36]
***ARH1***	▪ Tumor suppressor	▪ Poor prognosis in BC	▪ Hypermethylation in BC patients	[37]
***14-3-3 sigma***	▪ DNA damage repair	▪ Increases malignant transformation	▪ Hypermethylation in BC patients	[37]
***RIZ1***	▪ Tumor suppressor	▪ Inhibits apoptosis	▪ Hypermethylation in BC patients	[37]
***DAPK1***	▪ Tumor suppressor	▪ BC specific mortality increases	▪ Hypermethylation in BC patients	[38]

These differences in epigenetic patterns may predispose AA and EA women to specific BC subtypes or affect disease outcomes. As such, DNA methylation patterns may serve as diagnostic markers or help guide personalized treatment of different BC subtypes in individuals with a particular ancestry.

**Table 3:** Hypermethylated genes (other than TSGs) in patients with BC (reported data, race wise difference in methylation pattern not studied).

Genes	Regular Function	Effect after Promoter Hypermethylation	Comments	References
***CXCL12***	▪ Metastasis	▪ Increase in metastasis ▪ Poor prognosis in BC	▪ Hypermethylated in highly aggressive BC cell lines	[39]
***CXCR4***	▪ Cell–cell adhesion ▪ Cell proliferation and migration	▪ Better overall and disease-free survival	▪ Hypermethylated in lower grade and stage of BC	[40]
***TOX***	▪ DNA damage repair	▪ Poor prognosis in BC	▪ Hypermethylated in patients with BC	[41]
***SPOCK2***	▪ Invasion and metastasis	▪ Poor prognosis in BC	▪ Hypermethylated in BC cell lines	[33]
***DPY5***	▪ Involved in drug catabolism	▪ Poor prognosis in BC	▪ Hypermethylated in luminal A and B BC subtype	[33]
***PITX2***	▪ Useful marker in predicting the chemotherapeutic response	▪ Poor prognosis in BC	▪ Hypermethylation in patients with triple negative breast cancer	[42]
***TP53***	▪ Induces apoptosis	▪ Worst outcome in BC ▪ Increases BC progression	▪ Hypermethylated in patients with BC	[43]
***MDM2***	▪ Promotes invasion and migration	▪ Better overall and disease-free survival	▪ Hypermethylated in lower grade and stage of BC	[28]

## 4. Differentially Methylated Genes in AA and EA Breast Cancer Patients: Unpublished Data from In Silico Analysis

**Figure 3:**
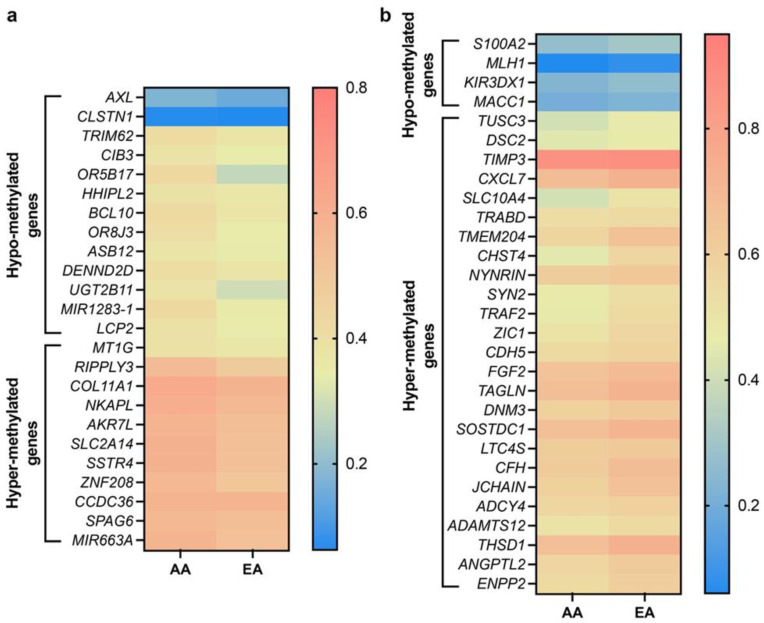
Heatmaps showing differential methylation pattern of various genes in AA and EA BC patients. (**a**) Heatmap showing significantly hypo- and hyper-methylated genes higher in AA over EA BC patients. (**b**) Heatmap showing significantly hypo- and hyper-methylated genes higher in EA over AA BC patients. AA, *n* = 160, EA, *n* = 578, *p* ≤ 0.05. Data were analyzed using graph pad prism 8 software.

We performed an in silico analysis of data obtained from 738 BC patients (AAs, *n* = 160; EAs, *n* = 578) to identify differences in methylation patterns between AA and EA BC patients using the UALCAN portal [44]. We determined the methylation status of genes regulating various functions in BC based on the β-value ranging from 0 (unmethylated) to 1 (fully methylated). Different β-value cutoffs have been considered to indicate hypermethylation (β-value: 0.5–0.7) or hypomethylation (β-value: 0.25–0.3). Out of ~150 genes, 50 genes were found to be significantly (*p* ≤ 0.05) differentially methylated between AA and EA BC patients; 36 genes were hypermethylated, and 14 were hypomethylated (Figure 3a,b, Table 4 and Table 5). Notably, hypermethylation of most TSGs (including *MIR663A*, *ZNF208*, and *NKAPL*) was more profound in AAs than in EAs. Furthermore, the TSG *S100A2* was hypomethylated in EA patients with BC. TSG hypermethylation in AA patients may contribute to aggressive tumor phenotypes and poor disease outcomes.

However, some TSGs were hypermethylated in EAs or hypomethylated in AAs. The methylation levels of the TSGs *TIMP3* and *TUSC3* were higher in EA than in AA patients, whereas *DENDD2D* and *TRIM62* were hypomethylated in AA patients with BC. The methylation levels of genes involved in tumorigenesis, metastasis, invasion, angiogenesis, and cell proliferation (e.g., *ENPP2*, *ANGPTL2*, *THSD1*, *LTC4S*, *DNM3*, *TAGLN*, *FGF2*, *CDH5*, *ZIC1*, *TANK/TRAF2*, *CXCL7*) were higher in EA patients with BC than in their AA counterparts. These differences in methylation patterns may contribute to the more aggressive disease course observed in AA BC patients than EA women. Although these differential methylation patterns may contribute to racial disparities in BC outcomes, further in vitro validation of these data is necessary.

## 5. Plausible Role of Histone Modifications in Breast Cancer Racial Disparity

Gene silencing by DNA hypermethylation is closely related to chromatin modifications involving acetylation, methylation, phosphorylation, sumoylation, deamination, proline isomerization, and ubiquitination. These chromatin modifications play a substantial role in the organization of DNA in nucleosomes [72]. Alterations in chromatin remodeling factors and histone-modifying enzymes are frequently observed in BC [73]. Histone modifications, along with DNA methylation, modulate the transcriptional state of many genes regulating cancer progression [73]. In general, histone acetylation, methylation, phosphorylation, and ubiquitination are associated with gene activation. In contrast, histone methylation, ubiquitination, sumoylation, deamination, and proline isomerization tend to repress gene expression. However, the role of these modifications in repressing or activating gene expression is context-dependent [74].

Although distinct histone modification patterns have been identified in BC samples and BC subtypes, these patterns have not been studied in racially diverse patients. Alteration in the methylation levels of histone 3, lysine 27 (H3K27me), a modification associated with gene repression, is a hallmark of transformation in BC [75]. In vitro and in vivo data suggest that H3K27me may be a suitable target for anti-neoplastic therapy [75]. Differential expression of H3K27me3 (trimethylation of H3K27) has also been reported among the different BC subtypes [76]. Holm et al. reported higher H3K27me3 levels in luminal A, HER2-enriched, and normal-like tumors than in basal-like, triple-negative, and luminal B subtypes. Furthermore, higher H3K27Me3 levels have been associated with better BC outcomes [76]. Genome-wide sequencing studies have shown that H3K27me deregulation can result from mutations in the H3K27 methyltransferase complex consisting of PRC2, EZH2, and accessory proteins [77]. These mutations alter H3K27me function and are linked to poor clinical outcomes in cancer patients; however, their role in BC racial disparities merits further investigation.

Changes in the global methylation status of histone H3 lysine 4 (H3K4) [78] and enzymes regulating these modifications [79] are also associated with tumorigenesis. The presence of the H3K4me3 mark in the promoter of the *ERBB2* (i.e., HER2) gene is associated with HER2-overexpressing breast carcinomas [80]. HER2 overexpression is associated with an increased risk of all-cause mortality and BC-specific mortality in EA women with low Native American (NA) ancestry [81]. Messier et al. reported a global increase in H3K4 methylation (H3K4me) and acetylation (H3K4ac) in BC cell lines relative to normal breast epithelial cells. An increase in H3K4ac marks was associated with early stage cancer progression. On the other hand, H3K4me was predominant in the metastatic cell line MDA-MB-231, indicating its potential role in epithelial-mesenchymal transition in late-stage cancer [82]. Considering the importance of the H3K4me and H3K4ac mark in BC progression, its potential role in BC disparities between AAs and EAs should be investigated.

**Figure 4:**
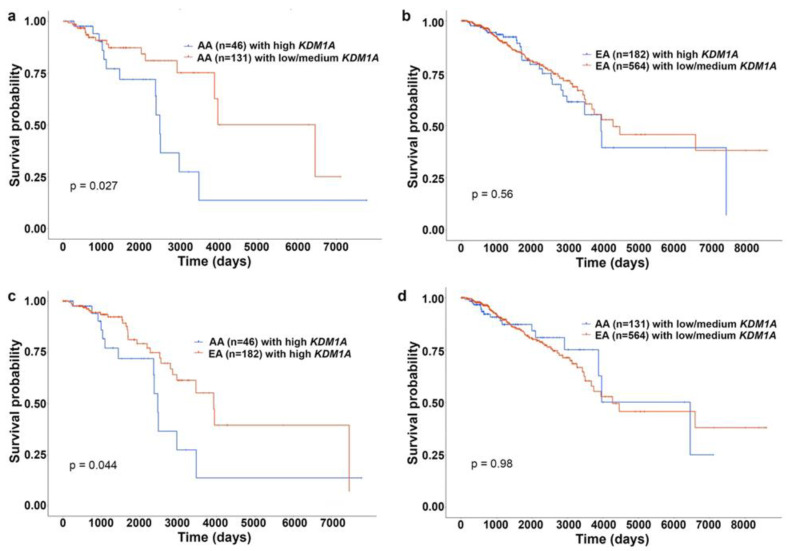
KM plots showing effect of *KDM1A* expression on survival of AA and EA patients with BC. (**a**) KM plot showing survival rates of AA patients with BC with high (blue) and low/medium (red) expression of *KDM1A*. (**b**) KM plot showing survival rates of EA patients with BC with high (blue) and low/medium (red) expression of *KDM1A*. (**c**) KM plot showing survival rates of AA (blue) EA (red) patients with BC with high expression of KDM1A. (**d**) KM plot showing survival rates of AA (blue) EA (red) patients with BC with low/medium expression of *KDM1A*.

Another chromatin-modifying enzyme involved in BC progression is lysine-specific demethylase 1(KDM1A). KDM1A catalyzes the removal of methylated groups from H3K4 and H3K9 [83]. Altered *KDM1A* expression is associated with tumorigenesis and is upregulated in various cancers [84]. KDM1A is involved in BC cell proliferation [85], invasion, and metastasis [86], and that *KDM1A* overexpression is associated with aggressive and poorly differentiated BC [83]. KDM1A also plays a critical role in BC chemoresistance by maintaining a pool of cancer stem cells [83]. Lim et al. identified KDM1A expression level as a predictive biomarker for aggressive tumor phenotypes biology in patients with BC, with ER- tumors exhibiting higher KDM1A expression levels [87]. Our survival analysis from UALCAN (AA, *n* = 46; EA, *n* = 182) confirmed the prognostic value of *KDM1A* expression level (Figure 4). For this analysis, samples were categorized into two groups: (1) High expression (with TPM values above upper quartile) and (2) Low/Medium expression (with TPM values below upper quartile).The Kaplan–Meier survival plot was generated for every gene in each TCGA cancer type, using “survival” package [88] and “survminer” package [89]. Our analysis revealed that high expression of KDM1A is associated with lower survival rates, particularly in AA than EA BC patients (Figure 4a–d). Thus, downstream genes epigenetically regulated by KDM1A may contribute to the aggressive BC phenotypes observed in AA patients. Variation in global levels of histone marks, such as lysine acetylation (H3K9ac, H3K18ac, H4K12ac, and H4K16ac), lysine methylation (H3K4me2 and H4K20me3), and arginine methylation (H4R3me2), are also linked with different BC subtypes and outcomes [20], but their role in racially diverse BC population is largely unexplored. Delineating these histone modifications and their effect on gene expression could help us understand the biological mechanisms underpinning BC racial disparities.

## 6. RNA Modifications—Another Layer of Epigenetic Regulation

The discovery of the first RNA modification enzyme, such as fat mass and obesity associated protein (FTO), which promotes oxidative demethylation of abundant N6-methyladenosine (m6A) residues in RNA, underscores the idea that alteration in RNA may serve as an epigenetic marker [90]. Gene regulation by miRNAs is another critical epigenetic event, and its role in cancer health disparities is evolving [91]. ncRNAs, a cluster of RNAs that do not encode a functional protein, have recently garnered attention of scientists for their role in regulation of gene expression through epigenetic network [92]. The hypermethylation of CpG islands near the miRNA genes causes transcription repression by epigenetic silencing. A study by Nassar FJ [93] performed a comparative miRNA microarray profile analysis of Lebanese and matched American BC patients. Their study demonstrated 21 exclusively dysregulated miRNA (miR-31, 362-3p, and 663) and 4 miRNA (miR-1288-star and 324-3p) with different expression pattern in Lebanese than American patients with BC. A study by Lewis K et al.,2018 [94] suggested that a combinatorial treatment with suberoylanilide (HDAC inhibitor) and epigallocatechin gallate (EGCG) (DNMT inhibitor) down-regulated the expression of oncogenic miRNA-221/222 in AA TNBC cell lines, such as MDA-MB-157 and HCC 1806. However, this study failed to incorporate EA TNBC cell lines and other BC specific AA and EA cell lines. Thus, a comprehensive miRNA profiling analysis is warranted in AA and EA patients with BC to understand the race signatures that are responsible for differential disease course and therapeutic outcomes.

Furthermore, an aberrant miRNA activity is also responsible for ectopic expression of human telomerase reverse transcriptase (hTERT) that regulates the proliferative span of cells. Various studies have reported that hTERT reactivation by aberrant miRNA expression is an early molecular event during BC neoplastic transformation and enhances genomic instability [95,96,97]. The miRNAs control hTERT expression by either directly acting on 3′-UTR/ORF of hTERT mRNA or indirectly intervening with other genes that control hTERT expression. Wu et al. have demonstrated that miRNA-4458 suppressed expression of hTERT in selected BC cell lines [98]. Hypermethylation of the CpG sites within the hTERT promoter regulatory region causes transcriptional activation, and hypomethylation enables transcription repression [99]. Apart from CpG methylation, hTERT expression is also regulated by histone modification. Various studies have reported a decrease in repressive H3K9me3 and H3K27me3 mark while an increase in activator marks, such as ac-H3, H3K9ac, and ac-H4 acetylation on the hTERT promoter in various cancer cell lines [100,101]. A thorough analysis of differential miRNA signatures and their hTERT regulation could help delineate the disparate racial burden in BC.

## 7. Role of Diet and Nutrition in Breast Cancer Racial Disparities through Epigenetic Modifications

Diet plays a significant role in BC development and progression [102]. For example, a Mediterranean diet rich in fiber and consisting of vegetables, fruits, fish, and soy can significantly reduce BC risk. The epidemiological link between BC and dietary factors is also supported by studies describing the impact of diet on methylation patterns of genes involved in cancer-related pathways [103].

DNA methyltransferases use methyl donor groups, including S-adenosyl methionine (SAM), to catalyze DNA methylation [104]. The production of SAM is dependent on dietary sources of methyl groups, such as foods containing methionine and folate (vitamin B9). The amino acid methionine is abundant in fish, meat, and dairy products, whereas folate is present in green leafy vegetables and fruits. Mikol et al. reported that folate and methionine deficiencies significantly altered DNA methylation patterns and induced liver cancer in rats in the absence of a carcinogen [105]. Although the evidence linking folate deficiency with an increased BC risk has been mixed [106], the data are more conclusive for BC subtypes. Recent population-based studies suggest that higher folate intake may lower the risk of ER−/PR− [107] and ER− BC [107]. Similarly, impaired folate absorption and metabolism due to alcohol consumption are associated with an increased risk of BC [108]. Although the evidence for an association between folate intake and overall BC risk remains inconclusive, folate intake may be especially critical in reducing the risk of BC in women who consume moderate-to-high alcohol levels [107]. Sustained deficiency of methyl-group-containing foods [109] or intake of high levels of methyl antagonists, such as alcohol [110], can result in aberrant methylation patterns. Christensen et al. reported changes in methylation patterns in breast tumor tissues, with increased folate intake associated with lower global hypomethylation and higher alcohol consumption linked with increased global hypomethylation [110]. They also found an association between folate intake and IL17RB, a protein implicated in BC development and progression [111].

Obesity is also associated with an increased risk of BC incidence and mortality [112]. Munsell et al. reported a positive association between obesity and risk of ER+/PR+ BC but not ER−/PR− BC [95]. Consistent with the relationship between obesity and BC, several studies have also revealed a positive association between dietary fat intake and BC risk [113]. Some of the effects of obesity and dietary behaviors on BC risk may be mediated by changes in DNA methylation patterns. Delgado-Cruzata et al. found that weight-loss interventions in overweight BC survivors could increase global methylation levels in long interspersed element-1 (LINE-1) [96]. However, the study did not investigate whether these interventions led to a reduction in BC recurrence. Several population-based studies in BC patients have also revealed a positive association between obesity and hypermethylation of tumor suppressor genes, such as *RASSF1* [97], *BRCA1* [97], *RARB* [114], *ECAD* [114], and CDKN2A [114].

Differences in dietary patterns and the consequent epigenetic changes may, in part, explain the racial disparities in BC occurrence and outcomes. Poor and minority communities do not have access to a healthy, fiber-rich diet due to the lack of access to supermarkets that sell affordable healthy and fresh food [115]. Instead, these neighborhoods tend to have access to cheaper high-fat food and alcohol [116]. Such areas, referred to as food deserts, are associated with higher rates of tobacco use, diabetes, and high BC mortality rates [117]. Compared with EAs, AAs are more likely to have a diet high in cholesterol and low in dietary fiber and folate [118]. Furthermore, AA women are less likely to engage in physical activity or meet the suggested physical activity guidelines [119]. The lack of access to healthy food and physical inactivity may contribute to the high risk of BC among AA women [119,120].

## 8. Potential Role of Psychosocial Stress in Racial Disparities in Breast Cancer through Epigenetic Changes

An association between psychosocial stress and BC has been investigated. Although there is evidence to suggest a positive correlation between exposure to stressors with BC incidence [121,122] and mortality, other studies showed a lack of such an association [123]. These mixed results may, in part, be attributed to methodological differences, including differences in the composition of the study population, study design, and criteria used for defining and evaluating stressful events. Although the association between BC and chronic stress is inconclusive, growing evidence from animal studies suggests a positive association between chronic stress and BC.

The term “allostasis” refers to the physiological process through which the body achieves stability in response to external physical and social stressors [124]. Acute physical or mental stressors activate the central stress axis involving the hypothalamic–pituitary–adrenal (HPA) axis and the sympathetic nervous system (SNS). Glucocorticoids released by the HPA axis and catecholamines released by the SNS mobilize physiological systems to help overcome the stressors and achieve homeostasis [125]. Although activation of the stress system in response to acute stressors can be beneficial, exposure to chronic stressors can result in the dysregulation of the stress system and the various downstream physiological processes involved in maintaining allostasis. Chronic stress (allostatic load) is associated with the development of multiple diseases, including cancer [126,127]. Dysregulation of the SNS and the HPA axis [128] due to chronic stress can modulate BC progression and are associated with poor survival outcomes. Although catecholamines and glucocorticoids can directly influence the tumor microenvironment, chronic stress can also promote inflammation, facilitating tumor progression [129]. Allostatic load has been used to quantify the cumulative physiological effect of exposure to stressors over an individual’s life course on multiple regulatory systems. Xing et al. found that prediagnostic allostatic load in AA women was associated with BC outcomes, tumor differentiation, and tumor size [130], highlighting the role of stress exposure in BC.

This role of allostatic load in BC outcomes may also contribute to racial disparities in BC. Compared with EAs, AAs, and other minority groups are more likely to experience multiple stressful events [131]. Studies have also shown that allostatic load is higher in AAs than in Eas [132], even after adjusting for socioeconomic status [133].

The role of social stressors in BC development and outcomes has received increasing attention in recent years. Social isolation is associated with high mortality in women with BC [134]. A population-based study showed that the absence of social ties was associated with a late-stage diagnosis of BC in AAs and higher mortality in both AAs and Eas [135].

Animal studies have shown that prenatal stress can impact the developing HPA axis, resulting in increased response to stressors (stress reactivity) in the offspring [136,137]. The changes in stress reactivity due to pre- and post-natal stress are related to epigenetic changes in brain regions involved in controlling the HPA axis [138]. The increased stress reactivity is marked by reduced glucocorticoid receptor (GR) expression in the hippocampus. Studies in rodents have shown that lower post-natal care results in increased methylation of the proximal promoter region of the GR gene, *NR3C1*, accompanied by lower GR expression in the hippocampus [138]. Changes in the methylation of the *NR3C1* promoter due to early life stress exposure have also been reported in human studies. Similarly, Conrad et al. showed that prenatal maternal depression was associated with increased methylation of *NR3C1* and *HSD11B2* in placental samples. In participants from the Black Women’s Health Study, the methylation status of the proximal promoter of *NR3C1* in adults was correlated with the frequency and severity of childhood sexual abuse [139]. These findings indicate the lasting impact of early life experiences on the HPA axis. Increased methylation of the *NR3C1* promoter is associated with unfavorable health outcomes, including BC [140] and depression [141]. Nesset et al. reported that the *NR3C1* proximal promoter was hypermethylated in 15% of BC samples, with most of these samples being ER+ tumors [140]. Hypermethylation of the *NR3C1* promoter was accompanied by lower GR expression in BC tissues, associated with poor survival outcomes in patients with ER+ BC [142].

Epigenetic changes have also been observed in stress- and immune-related genes due to exposure to stressors associated with residing in a disadvantaged neighborhood. For example, Needham et al. reported that low socioeconomic status in childhood was associated with hypermethylation of stress-associated genes (e.g., *AVP*, *FKBP5*, and *OXTR*) and inflammation-related genes (e.g., *CCL1* and *CD1D*) [143]. Smith et al. described methylation patterns in stress and inflammation-related genes associated with neighborhood socioeconomic disadvantage and neighborhood social environment during adulthood [144]. Neighborhood socioeconomic disadvantage was associated with the methylation of the stress-related genes (e.g., *CRF* and *SLC6A4*) and the inflammation-related genes (*F8* and *TLR1*). In contrast, an adverse neighborhood social environment, a measure encompassing social cohesion and safety, was associated with the methylation of stress-related genes (*AVP*, *BDNF*, *FKBP5*, and *SLC6A4*) and inflammation-related genes (*CCL1*, *CD1D*, *F8*, *KLRG1*, *NLRP12*, *SLAMF7*, and *TLR1*) [144]. Some of these stress and immune-related genes have been implicated in BC development and progression. Notably, *FKBP5* downregulation in BC is associated with chemoresistance [145], and *SLC6A4* (encoding the serotonin reuptake transporter) is essential for the activity of breast tumor-initiating cells [146]. Similarly, downregulation of *CD1D* in BC cells allows them to evade detection by natural killer T cells [147]. *CCL1* upregulation is associated with BC cells invasion [148]. These studies suggest that epigenetic changes mediated by differential exposure to psychosocial stress may contribute to racial disparities in BC.

## 9. Potential Role of Environmental Toxicants in Racial Disparities in Breast Cancer through Epigenetic Changes

Emerging evidence suggests that air pollutants can also increase the risk of BC [149]. Studies show that individuals residing in the proximity of chemical facilities and high-traffic urban zones are more susceptible to BC [150]. Traffic- and industry-related air pollutants, including nitrogen dioxide, sulfur dioxide, polycyclic aromatic hydrocarbons (PAHs), particulate matter, and carbon monoxide, are associated with an increased risk of BC [151]. PAHs [152] and metallic pollutants [153] exert their carcinogenic effects by mimicking or antagonizing the actions of estrogen and, thus, acting as endocrine disruptors. Emerging evidence also suggests that air pollutants may enhance the risk of BC through epigenetic mechanisms [149,154].

Individuals belonging to minority communities or having low socioeconomic status are at a disproportionately high risk of exposure to air pollutants. For example, AAs are more likely to inhabit localities in the vicinity of polluting industrial facilities [155]. These inequalities may be attributed to residential segregation along racial lines, with disenfranchised communities often having limited power to influence decisions where the industries are located [156]. Moreover, discriminatory zoning policies or those designed in the past with such intent often allow polluting industries to enter such neighborhoods. Furthermore, non-white, and low-income children are more likely to reside in high-traffic areas [157]. A recent prospective study showed that childhood residence in the proximity of a road with a median or other form of barrier suggestive of high vehicular traffic was associated with an increased risk of BC [158]. Thus, AAs are more likely to be exposed to air pollutants, and aberrant epigenetic alterations due to air pollutants may contribute to racial disparities in BC.

### 9.1. Metallic Pollutants

Although there are inconsistencies in the literature regarding the specific metallic pollutants associated with an elevated risk of BC, accumulating evidence suggests that long-term exposure to at least some of these pollutants can promote BC development. Recent studies showed that metallic air pollutants, including arsenic, lead, mercury, antimony, and cadmium, can increase the Torisk of BC [159,160]. The California Teachers Study revealed an association between cadmium and arsenic exposure and hormone receptor-negative BC among non-smokers [161]. Metallic air pollutants accumulating in the adipose tissue of the breast can have endocrine-disrupting effects [162]. Furthermore, the concentrations of cadmium, mercury, and lead in the breast tissue are higher in women with BC than in healthy women [162]. The study found that active concentrations of these metals are comparable to biomarker concentrations in AA women. In a methylomic analysis, Hanna et al. showed that lead and mercury exposure were associated with hypomethylation of the *COL1A2* promoter and hypermethylation of the *GSTM1* promoter, respectively [163]. Importantly, changes in *COL1A2* and *GSTM1* [164] expression have been associated with BC development and progression. These studies should further be extrapolated to racially diverse BC population to see if any difference in methylation patterns exist due to the higher concentration of metallic pollutants in AA than EA women.

### 9.2. Polycyclic Aromatic Hydrocarbons

PAHs are present in fossil fuel emissions, tobacco smoke, and smoked meats [165], and exposure to PAHs is linked to increased BC risk [166]. Furthermore, exposure to PAHs is associated with aberrant methylation patterns in promoters of cancer-related genes in BC cell lines and tissues. Exposure of the BC cell lines to the PAH benzo [a]pyrene (BaP) was associated with aberrant methylation of specific genes, but global methylation patterns remained unaltered [165]. Specifically, the TSG *TSC2* was significantly hypomethylated in MCF-7 cells exposed to BaP. Changes in TSC2 expression have been implicated in BC metastasis [167], suggesting that exposure to PAHs may affect BC outcomes due to aberrant DNA methylation. A population-based study demonstrated that polycyclic hydrocarbon exposure was associated with changes in the methylation status of various BC-related genes in peripheral blood cells. For example, synthetic log burning-related exposure to PAH was associated with hypermethylation of the tumor suppressor genes *HIN1* and *CDH1* and hypomethylation of the oncogene *CCND2*. In contrast, smoking and vehicular traffic exposure were associated with hypermethylation of the metastasis-related gene *TWIST1* and hypomethylation of the TSG *DAPK1* (encoding death-associated protein kinase 1) [166]. Callahan et al. investigated the association between lifetime PAH exposure and epigenetic changes in BC by estimating the exposure to particulate matter and traffic emissions (as a proxy for PAH) at the subject’s address at birth, menarche, and the birth of the first child [168]. They found a positive association between the methylation of BC-related genes, depending upon the source of PAHs and age at exposure. For example, exposure to traffic-related PAHs at birth was associated with increased methylation of the TSG *SYK* in breast tumor tissues [168]. To date there are no studies showing differential PAH exposure in AA and EA women with BC. However, such studies are warranted in future to understand disparate BC burden among AA and EA.

### 9.3. Nitrogen Dioxide

Nitrogen dioxide (NO_2_) is another air pollutant released by vehicles and industries as a by-product of fuel combustion. Exposure to NO_2_ in ambient air is associated with an increased risk of BC [169,170] and can alter methylation patterns in BC-related genes. Plusquin et al. investigated the association between lifelong exposure to air pollutants and methylation patterns in the blood samples of two cohorts from the European Prospective Investigation into Cancer and Nutrition (EPIC) study [171]. NO_2_ exposure was associated with global hypomethylation, especially at CpG island shelves and shores. The TSG *EPHB2* encoding ephrin type-B receptor 2 was among the significantly hypomethylated genes in response to NO_2_ exposure. Lower expression of EPHB2, (member of the Eph tyrosine kinase receptor family), is associated with tumor invasiveness, metastasis, and poor survival outcomes in BC [172,173]. Although these studies do not establish direct causation between various air pollutants and BC, they suggest that air pollutants may contribute to BC development and progression by inducing epigenetic changes.

## 10. Potential Role of Smoking in Racial Disparities in BC through Epigenetic Changes

Cigarettes contain a wide array of carcinogenic compounds and endocrine disruptors that can cause cancer [174]. Although the role of smoking in BC is inconclusive, recent studies revealed a modest association between chronic smoking and BC development [175,176]. Moreover, evidence exists that suggests that smoking may elevate the risk of ER+ BC but not of triple-negative BC [177,178]. In addition, smoking at the time of BC diagnosis has been associated with poor survival outcomes [179]. A large population-based study showed that smoking at the time of BC diagnosis was associated with higher BC mortality in the long term (13 years) but not in the short term (5 years) [180]. The study also showed that the negative impact of smoking on long-term survival was more profound in AA women than in EA women. Furthermore, smoking during BC diagnosis was associated with poor survival in women with ER- tumors but not in those with ER+ BC [180]. Although the prevalence of smoking is slightly higher in EA women than in AA women in the general population [181], a similar smoking rate has been reported at the time of BC diagnosis [180]. Therefore, racial differences in BC-associated mortality may be partly attributed to preexisting genetic variation and differences in ER status between AAs and EAs. However, it is also plausible that smoking could potentiate the effects of these genetic differences by modulating gene-environment interactions.

Epigenome-wide association studies showed that nearly 1000 CpG sites were differentially methylated in peripheral blood cells of active smokers and non-smokers [182]. Hypomethylation of CpG sites at the gene *AHRR* (encoding aryl hydrocarbon receptor repressor) and the intergenic locus 2q37.1 have been consistently observed in these studies [182]. In an epigenome-wide association study, Shenker et al. found that hypomethylation of 2q37.1 locus but not *AHRR* was associated with increased BC risk [183]. Furthermore, specific methylation patterns have been observed in breast tumors from smokers and non-smokers with different BC subtypes. Conway et al. reported that in hormone receptor-negative (HR−) tumors, hypomethylation of CpG sites in cancer-related genes was more profound in smokers than in non-smokers [184]. In contrast, smoking was associated with hypermethylation of CpG sites in BC-related genes in HR+ tumors [184]. Additionally, HR+ tumors from AA smokers showed higher hypermethylation levels compared with those from EA smokers. In HR- tumors, no differences in methylation levels were observed between AA and EA smokers.

Furthermore, the Carolina Breast Cancer study revealed significant racial differences in the methylation patterns of CpG sites in the promoter regions of several cancer-related genes in breast tumors [27]. Differentially methylated genes between AA and non-AA patients included the gene *AXL*, which was hypermethylated in tumor tissues from AAs. *AXL* encodes a receptor tyrosine kinase implicated in BC initiation and progression [185]. Notably, *AXL* hypermethylation has been associated with prenatal smoke exposure [186]. Chronic smoking has also been associated with lower oral [187] and serum folate levels [188]. In turn, folate deficiency can increase the risk of BC by impairing DNA methylation [189]. These smoking-associated differences in epigenetic patterns between AAs and EAs may contribute to racial disparities in BC incidence and clinical outcomes.

## 11. Conclusions

In this review article, we have outlined differences in epigenetic patterns between AA and EA patients with BC that may contribute to racial disparities in BC incidence and clinical outcomes. Despite evidence supporting the presence of racial differences in DNA methylation patterns in BC, such differences in histone modification patterns are underexplored. Some of the differentially methylated genes between AA and EA patients with BC have been identified as biomarkers of cancer development and progression. These genes could be used as diagnostic markers and to guide the development of personalized treatments. Differences in methylation patterns have also been observed between healthy AA and EA women. Although epigenetic regulation of genes can increase the risk of cancer in individuals of any race/ethnicity, AA women with higher basal epigenetic changes, might be disadvantaged and are, therefore, much more pre-disposed to cancer onset, and an aggressive disease course. The presence of preexisting racial differences in epigenetic profiles of breast tissues indicates that these profiles could be used for early diagnosis. Classification of the different BC subtypes in AA and EA BC patients with the help of detailed epigenetic profiles may allow for early diagnosis, prediction of prognosis, and development of personalized treatments. Characterizing the epigenetic patterns associated with BC in AA and EA patients may also improve our better understanding of the complex tumor biology of BC. Most current epigenetic profiling approaches involve the analysis of bulk tumor cells consisting of a diverse cell population. Thus, the use of epigenetic profiling as a tool for understanding tumor biology would require more fine-tuned approaches to capture the heterogeneity of cells populating tumors and the tumor microenvironment. Epigenetic changes due to social and environmental factors may also mediate racial disparities in BC outcomes. Differences in dietary habits, exposure to environmental pollutants, and psychosocial stress associated with elevated BC risk are, to a large extent, a consequence of racial and economic inequalities. Although it may be tempting to suggest mitigating measures on the community or local level to reduce BC disparities, the structural inequalities at the core of these disparities necessitate urgent broader systemic changes that complement local action.

## Figures and Tables

**Table 1:** Hypermethylated TSGs in racially diverse AA and EA patients with BC (reported data).

Tumor Suppressor Genes	Regular Function	Effect after Hypermethylation	Comments	References
***RARB***	▪ Promotes apoptotic cell death	▪ Increase in BC progression ▪ Increase in metastasis	▪ Hypermethylation in AA compared to EA BC patients	[24]
***CDH13***	▪ Cell–cell adhesion ▪ Inhibits cell invasion and proliferation	▪ Increases risk of BC	▪ Hypermethylation in ER negative AA patients with BC than EA. However, not in ER positive AA and EA BC patients	[24]
***RASSF1***	▪ Tumor suppressor	▪ Poor outcome in BC ▪ Increase in metastasis	▪ Hypermethylation in AA than EA BC patients	[24]
***SCGB3A***	▪ Regulates cell proliferation and differentiation	▪ Increase in metastasis ▪ Poor outcome in BC	▪ Hypermethylation in AA than EA BC patients	[21]
***TWIST1***	▪ Promotes invasion and metastasis	▪ Increase in metastasis ▪ Poor outcome in BC	▪ Hypermethylation in AA than EA BC patients	[21,25]
***CCND1***	▪ Induces senescence like phenotype ▪ Inhibits cell proliferation	▪ Increases BC risk ▪ Increase in metastasis	▪ Hypermethylation in AA than EA BC patients	[21]
***SFRP1***	▪ Negative regulator of WNT	▪ Increases BC progression	▪ Hypermethylation in older AA than older EA BC patients	[26]
***TUSC3***	▪ Tumor suppressor	▪ Poor survival	▪ Hypermethylation in AA than EA BC patients leading to poor survival in AA	[27]
***TES***	▪ Tumor suppressor	▪ Poor survival	▪ Hypermethylation in AA than EA BC patients leading to poor survival in AA	[27]

**Table 4:** Hypermethylated genes in AA and EA patients with BC (in silico analysis, unpublished data).

Genes	*p* Value	Hypermethylation Higher in	Reported Function in Cancer	Reference
***ENPP2***	0.00456	EA	▪ Encodes autotaxin which mediates mammary tumorigenesis and cancer cell migration	[45,46]
***SPAG6***	0.0060803	AA	▪ Function not documented	NA
***ADAMTS12***	0.03684	EA	▪ Exhibits both oncogenic and tumor-suppressive effects ▪ Acts as an angio-inhibitory proteinase with the ability to confer anti-tumorigenic properties to epithelial or endothelial cells	[45,46]
***CCDC36***	0.00326	AA	▪ Function not documented	NA
***ADCY4***	0.00514	EA	▪ High levels of ADCY4 are associated with better prognosis in patients with BC	[47]
***JCHAIN***	0.0104	EA	▪ Function not documented	NA
***ZNF208***	0.00058	AA	▪ Acts as a tumor suppressor	[48]
***CFH***	0.0073846	EA	▪ Downregulation of CFHR1 is associated with lower overall survival (OS) and post progression survival (PPS) times	[45,46]
***SSTR4***	9.94 × 10^−5^	AA	▪ Induces cell cycle arrest	[49]
***LTC4S***	0.01162	EA	▪ Associated with tumor aggressiveness	[50]
***SLC2A14***	2.00 × 10^−5^	AA	▪ Satisfies the high metabolic demands of cancer cells	[45,46]
***SOSTDC1***	0.00166	EA	▪ SOSTDC1 is expressed in normal breast tissue and this expression is reduced in breast cancer ▪ High levels of SOSTDC1 mRNA correlated with increased patient survival conversely, SOSTDC1 protein levels decreased as tumor size and disease stage increased	[51,52]
***DNM3***	0.0118419	EA	▪ The stable silencing of DNM3 showed a trend in reduced cell migration compared to control shRNA ▪ DNM3 expression showed a significant inverse correlation to its metastasis suppressing genes (MSG) set	[51,52]
***TAGLN***	2.97 × 10^−6^	EA	▪ TAGLN positivity is associated with more aggressive tumors, high Ki-67 count and low ER and PR expression	[53]
***FGF2***	0.01886	EA	▪ Induces cell proliferation and tumor growth	[51,52]
***AKR7L***	0.00012	AA	▪ Function not documented	NA
***CDH5***	0.03354	EA	▪ CDH5 levels and its glycosylation represent biomarker that distinguish patients with metastatic breast cancer from those that remain metastasis-free	[51,52]
***ZIC1***	0.00666	EA	▪ Higher ZIC1 RNA expression indicates a better overall survival in the breast cancer samples	[54]
***TANK/TRAF2***	0.01394	EA	▪ Over expression of TRAF2 in human MDA-MB-231 BC cells increases cell growth and motility in vitro, whereas TRAF2 knockdown shows inhibitory effect	[55]
***NKAPL***	3.67 × 10^−6^	AA	▪ NKAPL may be involved in mechanism of cancer suppression in breast cancer tissues ▪ High methylation level of NKAPL and its low expression predicts poor outcome	[56]
***SYN2***	0.00164	EA	▪ Function not documented	NA
***NYNRIN***	0.00022	AA	▪ Function not documented	NA
***CHST4***	0.00358	EA	▪ Function not documented	NA
***COL11A1***	0.039	AA	▪ Overexpression leads to cancer cell proliferation, invasion, migration, and metastasis	[57]
***TMEM204***	3.70 × 10^−6^	EA	▪ Function not documented	NA
**TRABD**	0.01552	EA	▪ Function not documented	NA
***SLC10A4***	0.01166	EA	▪ Function not documented	NA
***RIPPLY3***	0.00072	AA	▪ Function not documented	NA
***PPBP/CXCL7***	0.0364	EA	▪ The CXCL7/CXCR2 axis may be important in breast cancer metastasis ▪ Therapeutics aimed at antagonizing CXCL7, may be beneficial in preventing invasion and, thus, the spread of BC	[58]
***TIMP3***	0.03268	EA	▪ TIMP-3 may have many anticancer properties, including apoptosis induction and antiproliferative, antiangiogenic, and antimetastatic activities	[59]
***DSC2***	0.00672	EA	▪ Loss of DSC2 promotes cell proliferation and enables tumor growth	[60]
***TUSC3***	3.47 × 10^−8^	EA	▪ A putative tumor suppressor gene ▪ Epigenetic silencing of TUSC3 has been associated with poor prognosis, and hypermethylation of its promoter provides an independent biomarker of overall and disease-free survival in ovarian cancer patients	[61]
***MT1G***	0.0378	AA	▪ MT1G hypermethylation showed a significant correlation with poor prognosis of patients with hepatoblastoma	[62]

**Table 5:** Hypomethylated genes in AA and EA patients with BC (in silico analysis, unpublished data).

Genes	*p* Value	Hypomethylation Higher in	Reported Function in Cancer	Reference
*MACC1*	0.00758	EA	▪ Increased serum MACC1 is associated with breast cancer TNM stage, tumor size, lymph node metastasis, and Ki-67 status ▪ MACC1 promotes BC cell proliferation and invasion	[63]
*KIR3DX1*	0.00452	EA	▪ Function not documented	NA
*LCP2*	0.01498	AA	▪ LCP2 functions in lymphatic vessel development ▪ LCP2 promotes T-cell development and activation	[64]
*MIR1283-1*	0.0185	AA	▪ Function not documented	NA
*UGT2B11*	3.97 × 10^−6^	AA	▪ Involved in metabolic processes	[65]
*DENND2D*	0.01004	AA	▪ Tumor suppressor gene	[66]
*ASB12*	0.032	AA	▪ Mediates the ubiquitination and subsequent proteasomal degradation of target proteins	The human protein atlas
*OR8J3*	0.03028	AA	▪ G-protein coupled receptor	The human protein atlas
*BCL10*	0.04469	AA	▪ BCL10 is involved in the formation of complexes that antagonize apoptosis and contribute to cell survival after DNA damage ▪ BCL10 is commonly involved in promoting the growth and invasion of cancer cells	[67]
*HHIPL2*	0.0261	AA	Involved in lipid cell metabolism	[51,52]
*OR5B17*	2.03 × 10^−5^	AA	2. Function not documented	NA
*CIB3*	0.00180324	AA	3. Function not documented	NA
*TRIM62*	0.0007	AA	4. TRIM62 is a regulator of cell polarity and a tumor suppressor in BC	[68]
*CLSTN1*	0.027603	AA	5. The alternative splicing of CLSTN1 predicts breast cancer patient survival	[51,52]
*MLH1*	0.04104	EA	6. Female MLH1 carriers would appear to be at moderate risk of BC and should be considered for breast screening at ages earlier than national screening programs	[69]
*AXL*	8.29 × 10^−5^	AA	7. AXL expression correlates with the acquisition of mesenchymal features of cancer cells and increases invasion 8. AXL expression in HER2+ breast cancers correlates with poor patient outcome	[70]
*S100A2*	0.0004414	EA	▪ The calcium-binding protein S100A2 is expressed in normal breast tissue but downregulated during breast cancer progression. ▪ A candidate tumor suppressor gene.	[71]

## Data Availability

The in-silico results of BC AA and EA samples shown here are in whole or part based upon data generated by the TCGA Research Network: https://www.cancer.gov/tcga (accessed on 22 January 2022). The data underlying this article will be shared on a reasonable request to the corresponding author (R.A.).
